# Supplementary figures and images for: The Plasmodium falciparum rhoptry bulb protein RAMA plays an essential role in rhoptry neck morphogenesis and host red blood cell invasion
Source: PLoS Pathog. 2019 Sep 6;15(9):e1008049. doi: 10.1371/journal.ppat.1008049 (PMC6750612; doi:10.1371/journal.ppat.1008049)

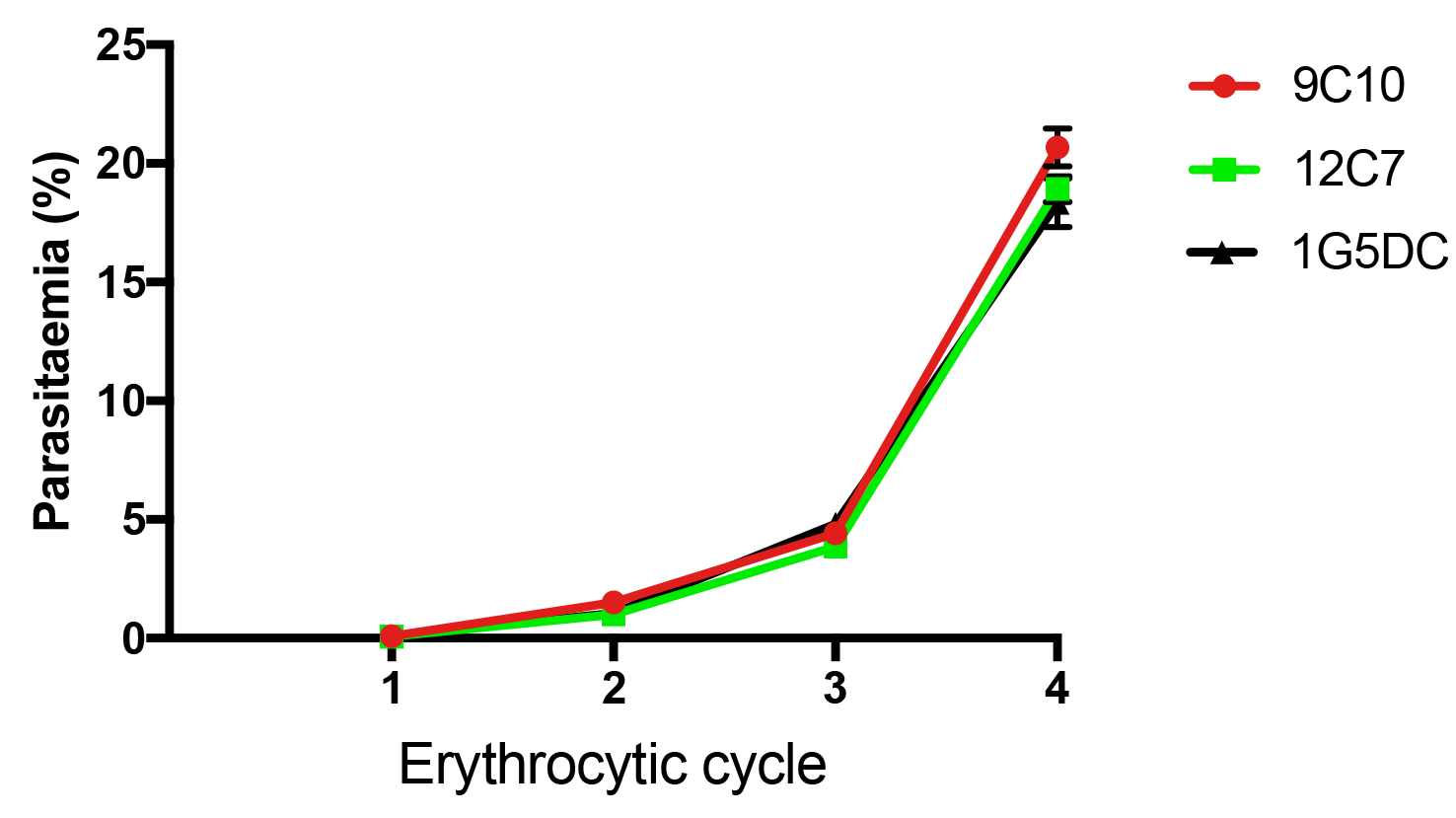

Supplement: S1 Fig — Parasitaemia values (quantified by flow cytometry) were averaged from three biological replicate experiments performed using blood from different donors, and are presented as mean ± SD. (TIF) [file ppat.1008049.s001.tif]

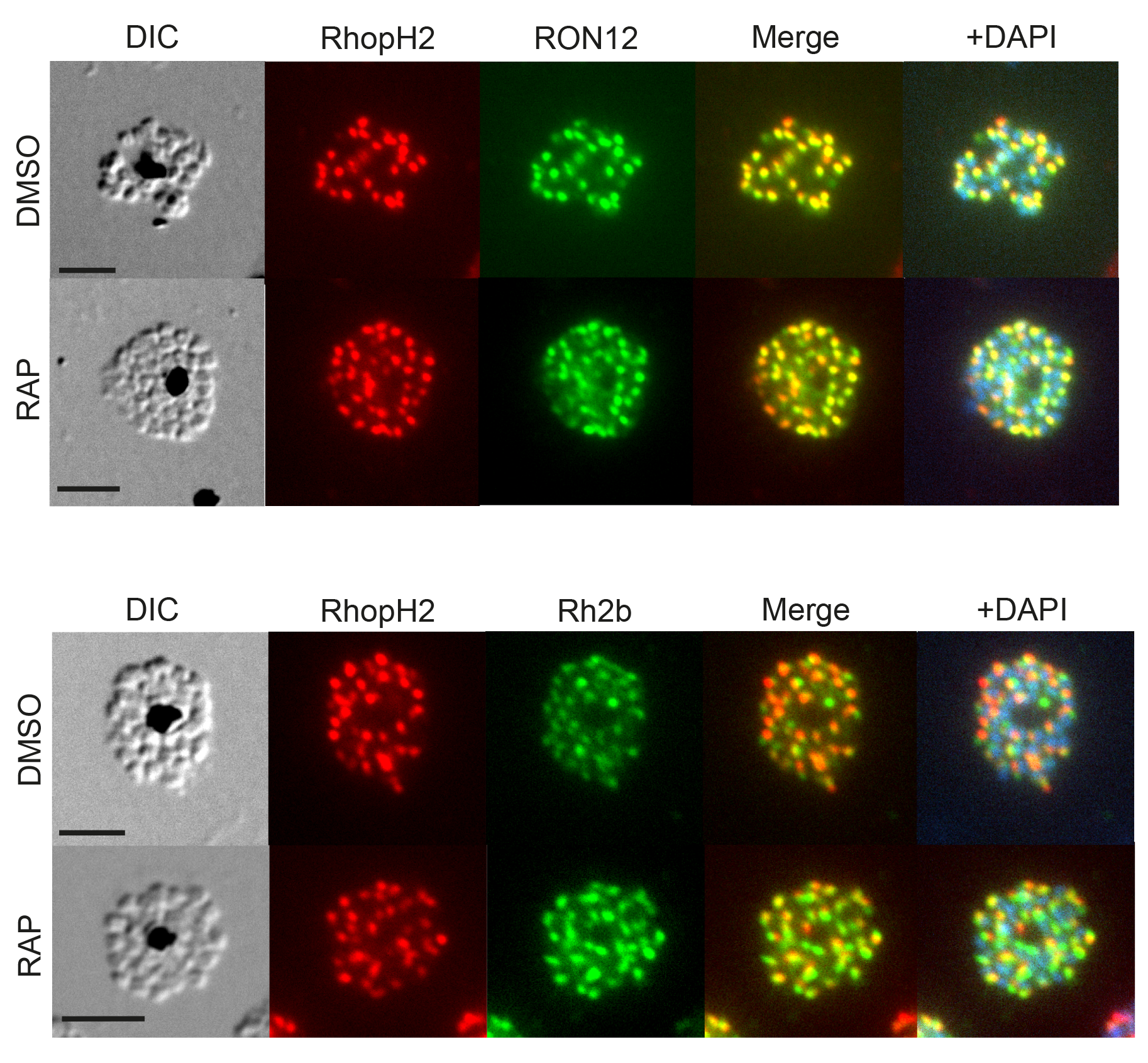

Supplement: S2 Fig — IFA showing that the staining profiles of both RON12 and Rh2b were unaltered between DMSO- and RAP-treated RAMAloxP clone 9C10 schizonts. Antibodies to RhopH2 were used as a marker for the rhoptry bulb. Scale bar, 5 μm. (TIF) [file ppat.1008049.s002.tif]
