# Supplementary material for: The Plasmodium falciparum rhoptry bulb protein RAMA plays an essential role in rhoptry neck morphogenesis and host red blood cell invasion
Source: PLoS Pathog. 2019 Sep 6;15(9):e1008049. doi: 10.1371/journal.ppat.1008049 (PMC6750612; doi:10.1371/journal.ppat.1008049)
Supplement: S1 Table — (PDF) [file ppat.1008049.s003.pdf]

S1 Table. Oligonucleotide primer sequences used in this study.

Primer name	Primer sequence (5' – 3')
RAMA_sgRNA_E2_F	attg**AGAATATGATTACGACGATA**[a]
RAMA_sgRNA_E2_R	aaacT**ATCGTCGTAATCATATTCT**
RAMA_sgRNA_E2S_F	attg**ATTACGACGATAAGGAATA**
RAMA_sgRNA_E2S_R	aaac**TATTCCTTATCGTCGTAAT**
RAMA_exon1_F (P11[b])	AATGGTATAAGCGGTCATTATACAGAAGGTAGG
RAMA_exon2recod_R (P13[b])	CCATACTCTTTGTCATCATAGTCGTATTCTTC
RAMA_exon2recod_F (P14[b])	GAAGAATACGACTATGATGACAAAGAGTATGG
RAMA_exon2_R (P15[b])	CTTCATCTTGTTGGACATCTAGAAGGTCTTC
RAMA_exon2WT_R (P12[b])	CTCTCCGTATTCCTTATCGTCGTAATC
DiCre_integrated	CTTTGCCATCCAGGCTGTTC
SERA5_DiCre_F	CAATATCATTTGAATCAAACAGTGGT
SERA5_DiCre_R	CCATTGGACTAGAACCTTCAT
RAMA_SB_homology2_F	GAAGAGATGAAATATGACGAGATGAAAGATG
RAMA_SB_homology2_R	AAGGTACTATGTAATTCATCGTCAGAATCTCC
RAMA_Harm1_F (P19[b])	TTATTGAGACAGACCATTATGAAAATAACG
RAMA_Harm2_R (P20[b])	ATCTTTCATCTCGTCATATTTCATCTCTTC

[a]Nucleotide sequences shown in bold represent sgRNA sequences.

[b]Numerical primer codes as used in Fig 1 and Fig 2 of the main manuscript.
